# Hiatal Hernia Mimicking Aortic Aneurysm on Point-of-care Echocardiography

**DOI:** 10.5811/cpcem.2017.6.33450

**Published:** 2017-10-03

**Authors:** Sam Langberg, Mark Favot

**Affiliations:** * Wayne State University School of Medicine, Department of Emergency Medicine, Detroit, Michigan; † Detroit Receiving Hospital, Department of Emergency Medicine, Detroit, Michigan; ‡ Sinai Grace Hospital, Department of Emergency Medicine, Detroit, Michigan

## CASE PRESENTATION

An 85-year-old woman presented to the emergency department (ED) with altered mental status. She appeared to be in shock with a distended abdomen. A point-of-care (POC) echocardiogram using a 4 Mhz phased array transducer revealed a large anechoic mass posterior to the left atrium concerning for an aneurysm of the descending thoracic aorta (DTA). (Image, Video) However, computed tomography revealed high-grade small bowel obstruction, associated with a hiatal hernia.

## DIAGNOSIS

The detection of hiatal hernia on echocardiography has been described in the cardiology literature;^1^ however, this case highlights a patient in shock who was diagnosed by POC echocardiography by emergency physicians (EP). Given the increased use of POC echocardiography by EPs, it is important to recognize mimics of life-threatening conditions. In the Image, a parasternal long-axis (PLAX) view reveals an anechoic mass posterior to the left atrium with multiple hyperechoic echoes within it, which raised suspicion for a DTA aneurysm. Other critical diagnoses in this anatomic region include aortic dissection, loculated pericardial effusion, left atrial or ventricular aneurysms.

When suspicious for a hiatal hernia on echocardiography, be certain to visualize the object of interest in at least two windows. The inner lining of the structure should be thick (6–13mm) and resemble stomach mucosa with the presence of microbubbles.^2^ A diagnosis may be confirmed after having the patient drink a carbonated beverage, which will result in increased microbubbles and swirling echo densities.^3^ Other mass lesions seen adjacent to the DTA include left atrial myxomas or thrombosis, mediastinal hematomas or tumors, pericardial cysts and esophageal carcinoma.^4^

In conclusion, a posterior anechoic mass seen on the PLAX view on POC echocardiography can be mistaken for a DTA aneurysm, but may be accurately diagnosed as an hiatal hernia if it has a thick inner lining resembling stomach mucosa and contains microbubbles.

**CPC-EM Capsule**

What do we already know about this clinical entity?

Emergency physicians rely on point-of-care echocardiography for rapid detection of critical diagnoses such as aortic aneurysms and dissections.

What is the major impact of the image(s)?

A hiatal hernia may be distinguished from an aortic aneurysm on ultrasound by the presence of microbubbles and a thick inner lining resembling stomach mucosa.

How might this improve emergency medicine practice?

By recognizing mimics of life-threatening conditions on ultrasound, emergency physicians can better expedite patient care and resource utilization.

## Supplementary Information



## Figures and Tables

**Image:**
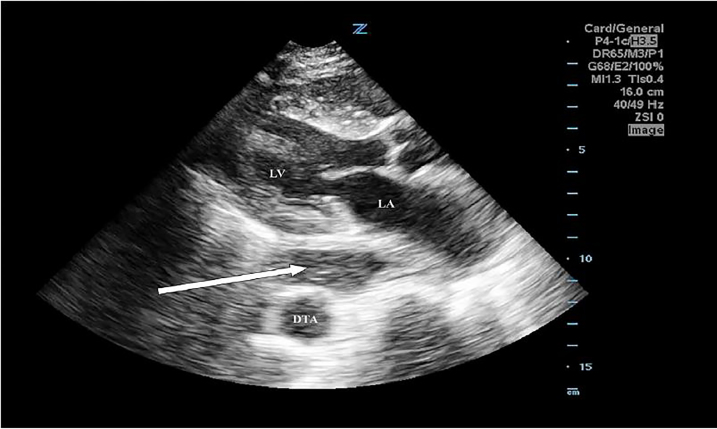
Parasternal long-axis window with anechoic mass (arrow) with microbubbles posterior to the left atrium (LA) and left ventricle (LV) but anterior to the descending thoracic aorta (DTA). Despite mimicking life-threatening aortic pathology, this is a fluid-filled hiatal hernia.
